# Clinical Outcomes and Mortality Following Delirium: A Five-Year Follow-Up Study on Hospitalized Patients

**DOI:** 10.3390/jcm15093453

**Published:** 2026-04-30

**Authors:** Ali M. Bahathig, Mohammed A. Alarabi, Muhammad H. Aldossary, Malak A. Almutairi, Mohammed A. Aljaffer, Ayedh H. Alghamdi, Fahad D. Alosaimi

**Affiliations:** 1 Department of Psychiatry, College of Medicine, King Saud University, Riyadh 11461, Saudi Arabia; 2 Department of Psychiatry, King Saud University Medical City, King Saud University, Riyadh 12372, Saudi Arabia; 3 SABIC Psychological Health Research and Applications Chair (SPHRAC), Department of Psychiatry, College of Medicine, King Saud University, Riyadh 11683, Saudi Arabia; 4 Eradah & Mental Health Complex, Riyadh 32272, Saudi Arabia

**Keywords:** delirium, mortality, outcomes, predictors

## Abstract

**Background:** Delirium is a common neuropsychiatric syndrome among hospitalized patients and has been associated with increased short- and long-term mortality. However, data on long-term outcomes and prognostic significance remain limited, particularly in Middle Eastern populations. **Methods:** This prospective observational cohort study was conducted at a tertiary care hospital in Riyadh, Saudi Arabia. Adult patients admitted to medical, surgical, and intensive care units (ICUs) underwent standardized clinical neuropsychiatric assessment for delirium. Five-year follow-up data were obtained from electronic health records and phone follow-up with patients/caregivers. Clinical outcomes and survival were compared between patients with and without baseline delirium. Kaplan–Meier survival analysis assessed five-year survival, and Cox regression explored the association between delirium and time-to-death adjusting for medical/psychiatric morbidity. **Results**: Among 278 patients, 71 (25.5%) were diagnosed with delirium during initial hospitalization. At five years, 51 patients (18.3%) had died. Patients with delirium had higher mortality (35.2% vs. 12.6%, *p* < 0.001), reduced five-year survival, and greater cumulative ward and ICU stays. In Cox regression including 50 deaths with complete data, delirium at initial assessment was associated with a higher hazard of death during follow-up (HR 1.93, 95% CI 1.06–3.55). Older age per year (HR 1.02, 95% CI 1.00–1.04), kidney disease (HR 2.12, 95% CI 1.20–3.75), and psychiatric disorders (HR 2.16, 95% CI 1.17–3.97) were independently associated with a higher hazard of death. **Conclusions**: Delirium was associated with increased five-year mortality and adverse long-term clinical outcomes. These findings support the prognostic significance of delirium in hospitalized patients.

## 1. Introduction

Delirium is an acute neuropsychiatric syndrome that is characterized by a sudden onset of confusion, impaired attention, and fluctuating cognitive function [1]. It commonly occurs in hospitalized patients, especially in the intensive care unit (ICU), and has been recognized as a marker of poor prognosis. The prevalence of delirium varies across the literature, between different in-hospital settings, and between different patient populations, reflecting its multifactorial nature and diverse clinical manifestations across medical and surgical contexts. In general medical settings, a meta-analysis found delirium prevalence of 23% among hospitalized older adults [2]. According to a systematic review and meta-analysis, the estimated prevalence of delirium in critical care settings is approximately 32%, with hypoactive delirium being the most common subtype, accounting for 45% of all delirium cases [3]. Many predisposing factors have been associated with increased vulnerability to delirium, such as advanced age, pre-existing cognitive impairment, functional limitations, reduced mobility, and sensory deficits [4,5,6,7]. Also, chronic medical conditions, particularly neurological and cardiovascular disorders like prior strokes, epilepsy, and heart disease, have been shown to heighten the risk of delirium significantly [4,8,9]. Furthermore, psychological and environmental factors, including prior hospitalization, long-term hospitalization, depression, and substance use, may also contribute to an individual’s susceptibility [4,10,11,12].

Delirium has been consistently associated with increased risk of mortality among hospitalized patients. Data from several meta-analyses show that elderly patients who develop delirium have two- to five-fold higher odds of death compared to those without delirium [13,14,15]. Mortality risk is highest during hospitalization and the early post-discharge period, but remains elevated at one year and persists for up to five years [13]. The risk was found to persist in another meta-analysis that included patients older than 16 years of age [16]. This supports the hypothesis that delirium may not simply be an acute cognitive disturbance but may also be a marker for mortality. Beyond mortality, delirium has been strongly associated with a range of adverse post-hospital outcomes. Meta-analyses consistently show that older patients who experience delirium have prolonged hospital and ICU stays, leading to increased health care utilization, including higher rates of unplanned readmissions in the months following discharge [13,14,15]. Studies showed that delirium in elderly patients can predict persistent cognitive impairment and functional decline [17,18], which can increase the burden on patients and their caregivers as well.

While much of the available evidence has been derived from Western populations, emerging data from the Middle East and North Africa region indicate that delirium is also highly prevalent and clinically significant in this context. A Saudi multicenter study across 14 ICUs reported a delirium prevalence of 45.9% among critically ill patients and highlighted the need for further research on long-term delirium outcomes in the region [19]. Similarly, a prospective cohort study from Oman reported a delirium prevalence of 55.4% among hospitalized older adults and identified several clinical risk factors associated with its occurrence [20]. Follow-up data from the same Omani cohort showed that delirium was associated with increased short-term and intermediate-term mortality, including at 90 days and one year [21]. Despite these important contributions, the regional literature remains limited in several respects. Follow-up has rarely extended beyond one year, and the extent to which delirium is independently associated with longer-term mortality after adjustment for confounding factors remains insufficiently studied. Therefore, interpretation of the existing literature is limited. Fewer studies employed the time-to-event method to define survival trajectories post-delirium, limiting insight into the timing of mortality risk. Additionally, there is a lack of data on long-term outcomes following delirium from the Middle East, hindering the generalizability of findings obtained mainly from Western healthcare systems.

The present study addresses these gaps by examining five-year clinical and survival outcomes following delirium in a prospective cohort of hospitalized patients in Saudi Arabia. In addition to providing extended follow-up, this study evaluates predictors of mortality using multivariable analysis to better understand the relationship between delirium and long-term outcomes in a Middle Eastern population.

## 2. Methods

This study represents the second phase of a prospective cohort study conducted at King Saud University Medical City (KSUMC), Riyadh, Saudi Arabia, a 1000-bed tertiary care teaching hospital. The initial phase of this study, titled *Validation of the Stanford Proxy Test for Delirium (S-PTD) among Critical and Non-critical Patients*, aimed to validate the S-PTD tool against clinical neuropsychiatric assessments to accurately identify delirium [22]. The first phase included adult patients consecutively admitted to the intensive care unit (ICU), medical, and surgical wards at KSUMC between June 2016 and October 2017.

### 2.1. Study Design and Objectives

The second phase of this prospective observational cohort study focused on identifying the five-year clinical outcomes following an episode of delirium. Secondary objectives include assessing five-year survival outcomes following delirium, including overall survival and time-to-death, and identifying possible predictors of mortality at five years.

### 2.2. Study Setting and Population

**Figure 1:**
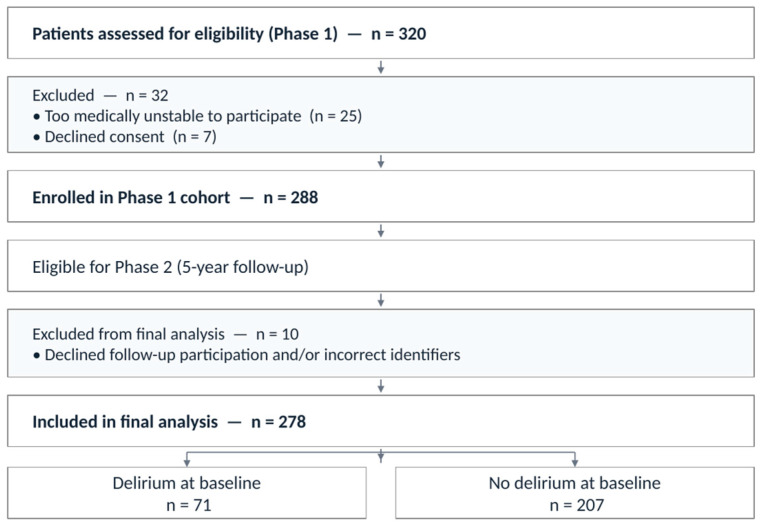
Flow Diagram of Patient Selection Across Study Phases.

The second phase targeted both groups (patients with/without delirium) included in the first phase of the study. The sample included patients who met the baseline inclusion criteria in the initial cohort and were screened for delirium. Eligible participants were adults aged 18 years and older admitted to the ICU, medical, and surgical wards during the initial phase, regardless of whether they were diagnosed with delirium on clinical neuropsychiatric assessment. No exclusions were made based on underlying medical, neurological, or psychiatric conditions, or on the use of medications associated with delirium risk. Accordingly, patients with pre-existing neurological disorders, including cognitive impairment and dementia, were included to reflect real-world clinical practice. Exclusion was limited to patients who were too medically unstable to participate in the initial assessment or for whom consent could not be obtained. Data from patients or their caregivers were gathered to provide updated information on the medical condition five years post-delirium diagnosis by phone. In the initial phase, 320 hospitalized patients were screened, of whom 288 met inclusion criteria and were enrolled. In the current follow-up phase, 278 patients were included in the final analysis after excluding individuals with missing or incorrect file numbers and those who declined participation in follow-up (Figure 1).

### 2.3. Data Collection

Data were collected from electronic health records (EHRs) and direct follow-up with patients or caregivers using the registered phone number between October 2020 and December 2021. Follow-up data relied mainly on EHRs, but also phone reports, using a structured data collection form, and included mortality status, chronic medical comorbidities, major surgical procedures, and diagnosed psychiatric disorders.

Medical comorbidity is represented both by individual diagnosis-specific variables, including cardiovascular, endocrine, renal, neurological, oncological, and other chronic conditions, and by a composite variable indicating the presence of two or more chronic medical conditions. Psychiatric morbidity is represented as a composite variable indicating the presence of any diagnosed psychiatric disorder, as well as specific diagnoses such as dementia, depressive disorders, anxiety disorders, psychotic disorders, bipolar disorder, trauma-related disorders, sleep disorders, and personality disorders also recorded when available. Surgical interventions were also collected, as well as the number of medications used and polypharmacy. These medical, surgical, psychiatric, and interventional variables were collected across the five-year period and therefore represent a cumulative burden of illnesses and interventions during follow-up.

Additional follow-up variables included the number of delirium episodes, chemotherapy sessions, radiotherapy sessions, hospital readmissions, ICU admissions, cumulative ward length of stay, cumulative ICU length of stay, and total duration of hospitalization.

### 2.4. Data Analysis

Data were analyzed using IBM SPSS Statistics, version 21 (IBM Corp., Armonk, NY, USA). Descriptive statistics are presented as means (M) or medians with standard deviations (SD) or interquartile range (IQR) for continuous variables, or as frequencies with percentages for categorical variables. The distributional characteristics of each variable were assessed through the Kolmogorov–Smirnov and Shapiro–Wilk tests, as well as skewness and kurtosis values. Given that most continuous variables were non-normally distributed, we used the Mann–Whitney U non-parametric test to investigate group differences in continuous variables. Categorical variables were compared using the Chi-square test of independence, with Fisher’s exact test applied when cell counts were below 5. The primary outcome was time to death over a five-year follow-up period, calculated in months from the initial assessment to the date of death or censoring. Patients who were alive at the end of follow-up were treated as censored observations. A Kaplan–Meier survival analysis with the log-rank test was used to assess five-year survival differences between patients with and without delirium.

To identify independent predictors of time-to-death, a Cox proportional hazards regression was performed. Given the limited number of death events, a parsimonious Cox proportional hazards model was specified a priori to reduce the risk of overfitting. Delirium at initial assessment was used as the primary predictor, while age was included as an essential variable. Amongst medical, psychiatric, and surgical comorbidities, we selected heart failure, kidney disease, and any diagnosed psychiatric disorder as indicative of medical/psychiatric morbidity. Variables potentially influenced by events during follow-up, such as cumulative ICU stay, were not included in the primary Cox regression model because they may lie on the causal pathway between delirium and mortality. The proportional hazards assumption was assessed through visual inspection of log-minus-log survival plots. No imputation was performed because missing data was infrequent. However, 4 cases were excluded from the survival model, including 1 deceased patient.

Statistical significance was defined at a two-tailed alpha level of 0.05, and values are presented with 95% confidence intervals.

### 2.5. Ethical Considerations

This study was conducted in accordance with the Declaration of Helsinki and approved by the Institutional Review Board of King Saud University Medical City (KSU Medical City), Riyadh, Saudi Arabia (E-15-1720; December 2015). All patients or their legal representatives who were enrolled provided informed consent for participation in the study. For follow-up data collection in the second phase, additional consent was obtained from patients or caregivers as needed to ensure continued ethical compliance.

## 3. Results

### 3.1. Sample Characteristics and Delirium at Initial Assessment

A total of 278 patients were included in the analysis, of whom 71 (25.5%) were diagnosed with delirium at initial assessment and 207 (74.5%) were not. Baseline characteristics and initial hospitalization variables are presented in Table 1. The mean age of the cohort was 57.7 years (SD = 18.48, 95% CI: 55.46–59.85), with a median of 58.5 years (range 22–98). Patients who experienced delirium during their initial hospitalization were older than those without delirium (mean 67.31 vs. 54.35 years; median 67.5 vs. 55.0), and this difference was significant (U = 4283.0, Z = −4.90, *p* < 0.001).

The mean duration of stay in hospital wards during the initial assessment was 20.0 days (SD = 36.47, 95% CI: 15.68–24.33), with a median of 9 days (range 0–365). The distribution was markedly right-skewed, indicating that most patients had brief ward stays while a minority remained hospitalized for prolonged periods. A similar pattern was observed for ICU stay, where the mean was 5.3 days (SD = 16.94, 95% CI: 3.32–7.33), with a median of 0 days (range 0–210), reflecting that most patients did not require ICU admission, while a minority had prolonged ICU stays. Compared with patients without delirium, those with delirium had significantly longer ward stays during the initial hospitalization (mean 25.00 vs. 17.86 days; median 12.5 vs. 8.0; U = 5943.0, Z = −2.31, *p* = 0.021) and significantly longer ICU stays (mean 7.71 vs. 4.17 days; median 1.0 vs. 0.0; U = 5459.0, Z = −3.64, *p* < 0.001).

### 3.2. Five-Year Survival Outcomes and Clinical Outcomes of Delirium

At the five-year follow-up, 51 patients (18.3%) were confirmed deceased, while 227 patients (81.7%) were alive. Among deceased patients (n = 51), the mean time to death was 20.1 months (SD = 17.05, 95% CI: 15.26–24.85), with a median of 13.0 months (range: 2.0–60.9). The distribution indicated that most deaths occurred within the first two years following the initial hospitalization, whereas a smaller number of deceased patients survived longer into the follow-up period. Table 2 shows the comparison of five-year clinical variables between patients with and without delirium at initial assessment and demonstrates that patients with delirium were more likely to have at least one diagnosed psychiatric disorder (*p* = 0.001). A more detailed comparison of medical, surgical, and psychiatric variables between the two groups is presented in Supplementary Table S1. In the detailed bivariate analysis, patients with delirium were more likely to have dementia, depressive disorders, and trauma-related disorders. Because most individual psychiatric diagnoses were infrequent, a composite variable indicating the presence of any diagnosed psychiatric disorder was used in further analyses.

**Figure 2:**
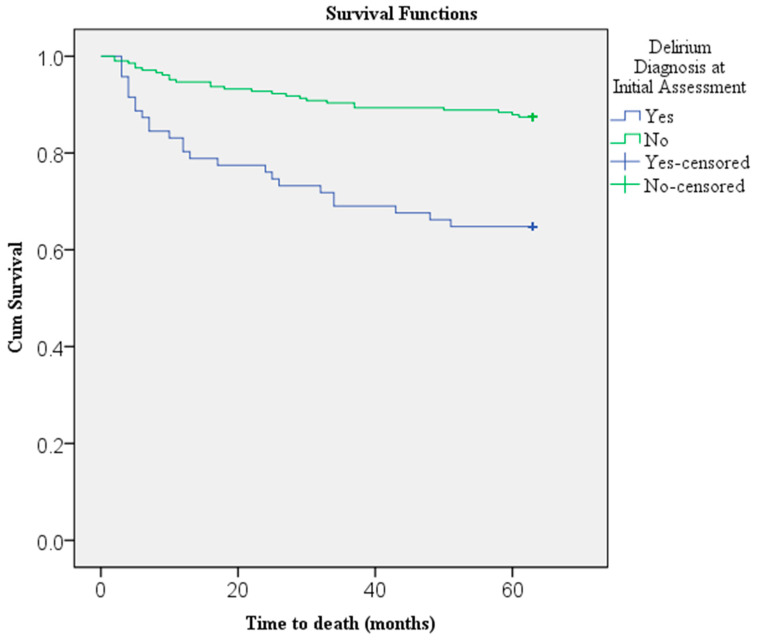
Kaplan–Meier Survival Curves Comparing Five-Year Mortality Between Patients with and Without Delirium at Initial Assessment (*N* = 278).

The Kaplan–Meier survival analysis demonstrated a significant difference in five-year survival between patients with and without delirium at initial assessment (Log-rank χ^2^ = 20.15, df = 1, *p* < 0.001) (Figure 2). At the end of the follow-up period, 64.8% of patients with delirium and 87.4% of those without delirium were alive. The mean survival time for patients who experienced delirium during their initial hospitalization was 46.89 months (SD = 23.73, 95% CI: 41.27–52.50; median = 62.98, IQR = 38.01), compared with 57.92 months (SD = 14.84, 95% CI: 55.89–59.95; median = 62.98, IQR = 0) for those without delirium. Although the median survival was similar between the two groups, the mean duration was notably shorter in the delirium group, and the difference was statistically significant (U = 5625.5, Z = −4.37, *p* < 0.001).

The number of hospital readmissions following the initial assessment ranged from 0 to 20, with a mean of 1.36 admissions (SD = 2.38, 95% CI: 1.08–1.65) and a median of 1 admission. Nearly half of the patients (47.8%) had no subsequent hospitalizations, while 25.2% were readmitted once, and the remaining patients experienced multiple readmissions. Patients who had experienced delirium during the initial hospitalization had a higher mean number of subsequent hospital admissions (M = 1.92, SD = 3.12, 95% CI: 1.15–2.70) compared to those without delirium (M = 1.18, SD = 2.07, 95% CI: 0.90–1.47); however, this difference did not reach statistical significance (U = 6008.0, Z = −1.41, *p* = 0.160). Comparisons of five-year clinical and procedural outcomes between patients with and without delirium are presented in Table 3. Patients who had delirium at the initial assessment demonstrated significantly greater cumulative ICU and ward stays, more recurrent delirium episodes, and a higher number of ICU admissions (*p* < 0.05).

### 3.3. Predictors of Mortality at Five-Year Follow-Up

In order to explore the predictors of time to death over the five-year follow-up period, we first examined the association of medical and psychiatric morbidity with mortality in bivariate analyses, which are detailed in Supplementary Table S2. Delirium at initial assessment was strongly associated with mortality in unadjusted analyses, with 35.2% of patients with delirium deceased compared to 12.6% without delirium (χ^2^ = 18.11, *p* < 0.001). Older age (U = 3593.5, Z = −3.96, *p* < 0.001) and longer cumulative ICU stay over the five-year period (U = 2827.5, Z = −5.96, *p* < 0.001) were also significantly associated with mortality. Similarly, the presence of multiple medical comorbidities, heart failure, kidney disease, and a diagnosed psychiatric disorder demonstrated significant associations with mortality.

A Cox proportional hazards regression model was used to examine predictors of time to death (in months) over the five-year follow-up period. Given the limited number of death events (n = 51), a parsimonious model was specified a priori to reduce the risk of overfitting. Delirium at initial assessment was used as the primary predictor, while age was included as an essential variable. Among the five-year medical, psychiatric, and surgical comorbidities, we selected heart failure, kidney disease, and any diagnosed psychiatric disorder as indicators of medical and psychiatric morbidity. All of these variables showed a significant association with mortality in bivariate analysis (Supplementary Table S2). Variables potentially influenced by events during follow-up, such as cumulative ICU stay, were not included in the primary Cox regression model because they may lie on the causal pathway between delirium and mortality. The proportional hazards assumption was assessed through visual inspection of log-minus-log survival plots (Supplementary Figure S1).

The overall Cox proportional hazards model is detailed in Table 4 and was statistically significant (χ^2^(5) = 44.48, *p* < 0.001). Of the total sample of 278 patients (and 51 deaths), a total of 274 had complete data and were included in the time-to-death Cox analysis, of whom 50 (18.2%) were deceased and 224 (81.8%) were censored. Delirium at initial assessment was independently associated with a higher hazard of death during follow-up compared with no delirium (HR = 1.93, *p* = 0.033). Increasing age per year was associated with a higher hazard of death over the follow-up period (HR = 1.02, *p* = 0.031). Kidney disease was also independently associated with a higher hazard of death (HR = 2.12, *p* = 0.009), as was the presence of any diagnosed psychiatric disorder (HR = 2.15, *p* = 0.013). Heart failure showed a non-significant trend toward a higher hazard of death over time (HR = 1.63, *p* = 0.103). Therefore, this model demonstrates that the association between delirium and mortality persisted after adjustment for age and predefined medical/psychiatric comorbidity of heart failure, kidney disease, and psychiatric disorders.

## 4. Discussion

In this study, delirium during the initial hospitalization was associated with adverse long-term outcomes, including reduced five-year survival and greater healthcare utilization. Delirium was strongly associated with mortality in unadjusted analyses and remained independently associated with a higher hazard of death, corresponding to a shorter time to death over follow-up, indicating that its prognostic effect was not fully explained by age, heart failure, kidney disease, or psychiatric comorbidity. In other words, after adjustment for age, heart failure, kidney disease, and psychiatric disorders, patients with delirium at initial assessment had nearly twice the risk of death at any given point during the five-year follow-up period. Beyond mortality, delirium was associated with sustained adverse clinical outcomes over five years. Patients with delirium had longer cumulative ward and ICU stays, more frequent ICU admissions, and recurrent episodes of delirium during follow-up. Although readmission rates did not differ significantly between groups, the overall pattern supports the established greater healthcare needs and illness burden among patients who develop delirium [15].

The observed association between delirium and increased mortality is consistent with prior studies and meta-analyses demonstrating that delirium identifies a population at heightened risk of death extending beyond the acute hospitalization [13,14,15]. In the present cohort, more than one-third of patients with delirium had died by five years, compared with approximately one in eight patients without delirium. Survival analysis further demonstrated a clear separation between groups, with most deaths occurring within the first two years following the initial hospitalization. This pattern is consistent with previous findings indicating that the increased mortality associated with delirium is most pronounced early after discharge but may persist for several years [13].

The identification of psychiatric comorbidity as an independent predictor of mortality over the five-year follow-up is a notable finding. Psychiatric comorbidity was associated with delirium at initial assessment but also independently associated with higher risk of mortality during the follow-up period. Patients with a documented psychiatric diagnosis had more than double the risk of death, even after adjustment for age, delirium, heart failure, and kidney disease. This association may be influenced by several factors, including underlying disease burden, reduced adherence to treatment, social vulnerability, and barriers to accessing ongoing care [23,24]. Psychiatric disorders may also interact with delirium through shared pathways such as cognitive impairment, medication burden, and stress-related physiological dysregulation [25]. Our bivariate analysis showed that dementia, depression, and trauma-related disorders were associated with delirium, as has been reported in the literature [7,11]. Although this study was not designed to investigate this association, these findings highlight the importance of considering mental health as a possible contributor to long-term outcomes in medically ill populations.

The consistency of our findings with reports from other settings suggests that the prognostic significance of delirium may be broadly similar across different socio-cultural contexts [26]. Furthermore, a recent systematic review also identified a wide range of predisposing and precipitating factors associated with delirium, further underscoring its marked pathophysiological heterogeneity [27]. This is relevant to our findings, which show that delirium can emerge in diverse clinical contexts, while mortality remains its most important adverse outcome. Taken together, these observations highlight the need for further work on both the predictors and outcomes of delirium. Given this heterogeneity, prevention should remain a clinical priority [28]. Our findings also support not only efforts to prevent delirium during hospitalization, but also closer post-discharge follow-up for patients who are diagnosed with delirium. This follow-up may include cognitive and functional reassessment, medication review, rehabilitation, and better integration of medical and psychiatric care. The focus on delirium prevention and follow-up may be even more relevant in the aftermath of the COVID-19 pandemic, during which delirium was frequently observed among hospitalized patients and was associated with poor clinical outcomes [29]. Emerging evidence on the cognitive and brain health effects of COVID-19 raises concern that the long-term burden of delirium, cognitive decline, and possibly dementia may increase since the pandemic, further highlighting the importance of prevention, early recognition, and longitudinal follow-up of delirium [30].

Our findings not only support the established link between delirium and mortality in general but also extend the regional literature by showing in a Saudi Arabian cohort that this association remains clinically relevant over five years and can be examined using adjusted survival analysis, beyond the largely unadjusted and shorter-term follow-up data currently available from the region. The strengths of this study include using a prospective cohort design, employing standardized delirium assessment with a clinical interview at baseline, and having an extended follow-up period. The use of survival analysis allowed characterization of mortality patterns over time, and the inclusion of clinically relevant covariates allowed for a more nuanced interpretation of the relationship between delirium and mortality. Additionally, data from a tertiary care center in the Middle East contribute to a literature that remains limited.

### Limitations

This study has several limitations that should be considered. It was conducted at a single center, which may limit generalizability to other healthcare settings. The inclusion of patients with diverse underlying neurological and medical conditions may have introduced heterogeneity that could not be fully accounted for in the analysis. Laboratory markers reflecting physiological stress or inflammation (e.g., C-reactive protein, neutrophil-to-lymphocyte ratio) were not consistently available and could not be included in the analysis. Data on delirium subtypes and management strategies were not systematically collected, limiting the ability to assess their potential influence on outcomes. The study did not stratify outcomes by admission type, whether medical or surgical, which may influence delirium risk and outcomes.

Another important limitation is that psychiatric morbidity was represented in our analysis by a composite variable indicating the presence of any diagnosed psychiatric disorder. This variable includes a heterogeneous range of conditions that may differ substantially in severity, chronicity, treatment exposure, and relationship to mortality. Therefore, while psychiatric disorders were independently associated with mortality in the adjusted model, the clinical interpretation of this association remains limited by the lack of diagnostic granularity. In addition, although dementia may have contributed to the observed association between psychiatric comorbidity and mortality, the small number of dementia cases makes it unlikely that dementia alone accounted for the finding.

Although the analyses accounted for several important covariates, residual confounding remains possible, such as the severity of the overall medical condition, which was used in previous studies but was not captured in our data. Several relevant baseline sociodemographic variables were not available, and the timing of follow-up diagnoses and procedures relative to delirium onset could not be established consistently. Therefore, including the cumulative five-year burden of medical and psychiatric comorbidities is a potential source of bias affecting the adjusted hazard ratios, limiting the interpretability of the results. Finally, the relatively small number of patients with delirium restricted the exploration of predictors within subgroups. Addressing these limitations in future research, particularly through multi-center studies, could provide a more comprehensive understanding of delirium’s impact on patient outcomes.

## 5. Conclusions

This prospective cohort study conducted at a tertiary care teaching hospital found that delirium in hospitalized patients was associated with adverse long-term outcomes, including reduced five-year survival, longer cumulative ward and ICU stays, more frequent ICU admissions, and recurrent episodes of delirium over five years of follow-up. After adjustment for age, heart failure, kidney disease, and diagnosed psychiatric disorder, delirium at initial assessment was associated with nearly twice the hazard of death at any given point during the five-year follow-up period. These findings support the prognostic significance of delirium and the need to recognize it as a clinical signal identifying patients who may benefit from closer follow-up and more integrated medical and psychiatric care after discharge. This study also highlights the need for multicenter research on long-term delirium outcomes in our region and on the impact of delirium prevention strategies.

## Figures and Tables

**Table 1:** Baseline Characteristics and Initial Hospitalization Variables for Patients With and Without Delirium at Initial Assessment (*N* = 278).

Variable	No Delirium (*n* = 207)	Delirium (*n* = 71)	*p*-Value
Age, mean ± SD	54.35 ± 17.89	67.31 ± 16.90	<0.001 *
Age, median (IQR)	55.0 (29.0)	67.5 (28.5)	
Initial ward stay (days), mean ± SD	17.86 ± 36.32	25.00 ± 36.88	0.021 *
Initial ward stay (days), median (IQR)	8.0 (13.5)	12.5 (24.0)	
Initial ICU stay (days), mean ± SD	4.17 ± 16.82	7.71 ± 15.44	<0.001 *
Initial ICU stay (days), median (IQR)	0.0 (3.0)	1.0 (9.8)	

*Note*: Continuous variables are presented as mean ± standard deviation and median (interquartile range). Between-group comparisons were conducted using the Mann–Whitney U test. * Statistically significant at *p* < 0.05. ICU, intensive care unit.

**Table 2:** Five-Year Clinical Variables for Patients With and Without Delirium at Initial Assessment (*N* = 278).

Clinical Variable		With Delirium n (%)	Without Delirium n (%)	*p*-Value
No. of patients		71	207	
Mortality status	Deceased	25 (35.2%)	26 (12.6%)	<0.001 *
	Alive	46 (64.8%)	181 (87.4%)	
≥2 chronic diseases	Yes	61 (85.9%)	156 (75.4%)	0.064
	No	10 (14.1%)	51 (24.6%)	
Any psychiatric disorder	Yes	23 (32.4%)	31 (15.0%)	0.001 *
	No	48 (67.6%)	176 (85.0%)	
Polypharmacy (≥5 medications)	Yes	24 (33.8%)	84 (40.6%)	0.312
	No	47 (66.2%)	123 (59.4%)	
Any major surgeries	Yes	36 (50.7%)	106 (51.2%)	0.942
	No	35 (49.3%)	101 (488%)	

*Note*: *p* values are two-sided Pearson chi-square tests. * Statistically significant at *p* < 0.05.

**Table 3:** Comparison of Five-Year Clinical Outcomes Between Patients With and Without Delirium at Initial Assessment (*N* = 278).

Five-Year Outcome Since Initial Assessment	With Delirium	Without Delirium	*p*-Value
No. of patients	71	207	
	**Mean ± SD (Median)**	**Mean ± SD (Median)**	
Number of hospital readmissions	1.92 ± 3.12 (1)	1.18 ± 2.07 (1)	0.160
Cumulative ward stay (in days)	719.68 ± 5287.06 (29)	35.24 ± 55.17 (16)	0.009 *
Cumulative ICU stay (in days)	16.94 ± 23.32 (7)	7.11 ± 20.38 (0)	<0.001 *
Number of delirium episodes	0.16 ± 0.44 (0)	0.05 ± 0.24 (0)	0.013 *
Number of ICU admissions	0.86 ± 1.22 (0.5)	0.48 ± 0.74 (0)	0.024 *
Number of hemodialysis sessions	3.59 ± 27.22 (0)	4.02 ± 54.75 (0)	0.004 *
Number of chemotherapy sessions	0.03 ± 0.17 (0)	0.84 ± 7.93 (0)	0.364
Number of radiotherapy sessions	0.04 ± 0.20 (0)	0.08 ± 0.62 (0)	0.905
Number of major surgeries	0.89 ± 1.23 (1)	0.84 ± 1.02 (1)	0.772

*Note*: Between-group comparisons were conducted using the Mann–Whitney U test. * Statistically significant at *p* < 0.05. ICU, intensive care unit.

**Table 4:** Cox Proportional Hazards Regression Model Predicting Time to Death Over Five-Year Follow-Up.

Covariate	Hazard Ratio (95% CI)	*p*-Value
Age (per year)	1.020 (1.002–1.039)	0.031
Delirium at initial assessment	1.934 (1.055–3.546)	0.033
Heart failure	1.634 (0.905–2.950)	0.103
Kidney disease	2.123 (1.203–3.745)	0.009
Any psychiatric disorder	2.155 (1.174–3.968)	0.013

*Note*: Model summary: χ^2^(5) = 44.48, *p* < 0.001.

## Data Availability

The data supporting the findings of this study are available within the article and its Supplementary Materials. Further inquiries can be directed to the corresponding author.

## Supplementary Materials

The following supporting information can be downloaded at: https://www.mdpi.com/article/10.3390/jcm15093453/s1, Table S1: Five-Year Medical, Psychiatric, and Surgical Comorbidities Among Patients With and Without Delirium at Initial Assessment (*N* = 278); Table S2: Bivariate Unadjusted Associations Between Clinical Variables and Mortality during 5-Year Follow-Up (*N* = 278); Figure S1: Log-Minus-Log Survival Plots for Patients With and Without Delirium at Initial Assessment.
