# Supplementary material for: Clinical Outcomes and Mortality Following Delirium: A Five-Year Follow-Up Study on Hospitalized Patients
Source: J Clin Med. 2026 Apr 30;15(9):3453. doi: 10.3390/jcm15093453 (PMC13164516; doi:10.3390/jcm15093453)
Supplement: Supplementary file 1 [file jcm-15-03453-s001.zip › jcm-4233067-supplementary.pdf]

Supplementary:

Table S1: Five-Year Medical, Psychiatric, and Surgical Comorbidities Among Patients With and Without Delirium at Initial Assessment (N = 278).

Comorbidity	Delirium		*p*-value
	**No (n=207)**	**Yes (n=71)**	
	Medical Comorbidities		
≥2 chronic diseases	156 (75.4%)	61 (85.9%)	0.064
Diabetes mellitus	97 (46.9%)	38 (53.5%)	0.333
Heart failure	38 (18.4%)	23 (32.4%)	0.014*
Hypertension	106 (51.2%)	48 (67.6%)	0.016*
Hyperlipidemia	57 (27.5%)	20 (28.2%)	0.918
Hypothyroidism	14/207 (6.8%)	12/70 (17.1%)	0.010*
Cerebrovascular disease	21 (10.1%)	17 (23.9%)	0.003*
Coronary artery disease	29 (14.0%)	14 (19.7%)	0.251
Kidney disease	49 (23.7%)	25 (35.2%)	0.058
Liver disease	10/207 (4.8%)	2/70 (2.9%)	0.483
Metastatic cancer	10 (4.8%)	2 (2.8%)	0.471
Non-metastatic cancer	14/207 (6.8%)	5/70 (7.1%)	0.914
Obesity	14 (6.8%)	2 (2.8%)	0.218
Old myocardial infarction	27/206 (13.1%)	11/71 (15.5%)	0.614
Osteoarthritis	5 (2.4%)	6 (8.5%)	0.024*
Neurological disease	26 (12.6%)	14 (19.7%)	0.138
Paralysis	0 (0.0%)	1 (1.4%)	0.087

Peptic ulcer disease	3 (1.4%)	4 (5.6%)	0.052
Pulmonary disease	41 (19.8%)	17 (23.9%)	0.459
Rheumatological disease	22 (10.6%)	6 (8.5%)	0.599
Seizure disorder	6 (2.9%)	14 (19.7%)	<0.001*
Psychiatric Comorbidities			
Any diagnosed psychiatric disorder	31 (15.0%)	23 (32.4%)	0.001*
Dementia	1 (0.5%)	5 (7.0%)	0.001*
Depressive disorder	15 (7.2%)	11 (15.5%)	0.039*
Anxiety disorder	10 (4.8%)	0 (0.0%)	0.059
Intellectual disability	0 (0.0%)	1 (1.4%)	0.087
Psychotic disorder	3 (1.4%)	2 (2.8%)	0.454
Bipolar disorder	4 (1.9%)	2 (2.8%)	0.658
OCD	0 (0.0%)	0 (0.0%)	-
Trauma-related disorder	0 (0.0%)	2 (2.8%)	0.015*
Eating disorders	0 (0.0%)	0 (0.0%)	-
Sleep disorders	4 (1.9%)	3 (4.2%)	0.287
Personality disorder	2 (1.0%)	1 (1.4%)	0.756
Surgical and Interventional Comorbidities			

Any major surgery	106 (51.2%)	36 (50.7%)	0.942
CABG	22 (10.6%)	8 (11.3%)	0.881
Heart valve replacement	6 (2.9%)	4 (5.6%)	0.286
Arterial bypass	3 (1.4%)	2 (2.8%)	0.454
Angioplasty/stent	23 (11.1%)	7 (9.9%)	0.769
Vein procedure	0 (0.0%)	1 (1.4%)	0.087
Hip fracture	1 (0.5%)	0 (0.0%)	0.557
Limb fracture	4 (1.9%)	1 (1.4%)	0.774
Total hip replacement	2 (1.0%)	1 (1.4%)	0.756
Total knee replacement	0 (0.0%)	2 (2.8%)	0.015*
Spine surgery	8 (3.9%)	1 (1.4%)	0.313
Foot amputation	5 (2.4%)	1 (1.4%)	0.614
Limb amputation	2 (1.0%)	1 (1.4%)	0.756
Appendectomy	3 (1.4%)	1 (1.4%)	0.980
Gallbladder surgery	10 (4.8%)	2 (2.8%)	0.471
Colon surgery	7 (3.4%)	1 (1.4%)	0.391
Stomach surgery	1 (0.5%)	1 (1.4%)	0.426
Breast surgery	1 (0.5%)	0 (0.0%)	0.557

Uterus/ovarian surgery	1 (0.5%)	2 (2.8%)	0.101
Prostate surgery	2 (1.0%)	1 (1.4%)	0.756
Brain surgery	9 (4.3%)	5 (7.0%)	0.370
Cancer-related procedure/condition	1 (0.5%)	0 (0.0%)	0.557

Note: p-values are from two-sided Pearson chi-square tests or Fisher's exact test for very sparse cells. * $p < 0.05$.

Supplementary Table S2. Bivariate Unadjusted Associations Between Clinical Variables and Mortality during 5-Year Follow-Up (n = 278).

**Variable**		**Alive (n = 227)**	**Deceased (n = 51)**	***p*-value**
Delirium at initial assessment	Yes	46 / 71 (64.8%)	25 / 71 (35.2%)	<0.001*
	No	181 / 207 (87.4%)	26 / 207 (12.6%)	
≥2 chronic diseases	Yes	169 / 217 (77.9%)	48 / 217 (22.1%)	0.002*
	No	58 / 61 (95.1%)	3 / 61 (4.9%)	
Diabetes mellitus	Yes	103 / 135 (76.3%)	32 / 135 (23.7%)	0.025*
	No	124 / 143 (86.7%)	19 / 143 (13.3%)	
Heart failure	Yes	41 / 61 (67.2%)	20 / 61 (32.8%)	0.001*
	No	186 / 217 (85.7%)	31 / 217 (14.3%)	
Kidney disease	Yes	49 / 74 (66.2%)	25 / 74 (33.8%)	<0.001*
	No	178 / 204 (87.3%)	26 / 204 (12.7%)	
Diagnosed psychiatric disorder	Yes	36 / 54 (66.7%)	18 / 54 (33.3%)	0.002*
	No	191 / 224 (85.3%)	33 / 224 (14.7%)	
Polypharmacy (≥5 medications)	Yes	106 / 108 (98.1%)	2 / 108 (1.9%)	<0.001*

	No	121 / 170 (71.2%)	49 / 170 (28.8%)

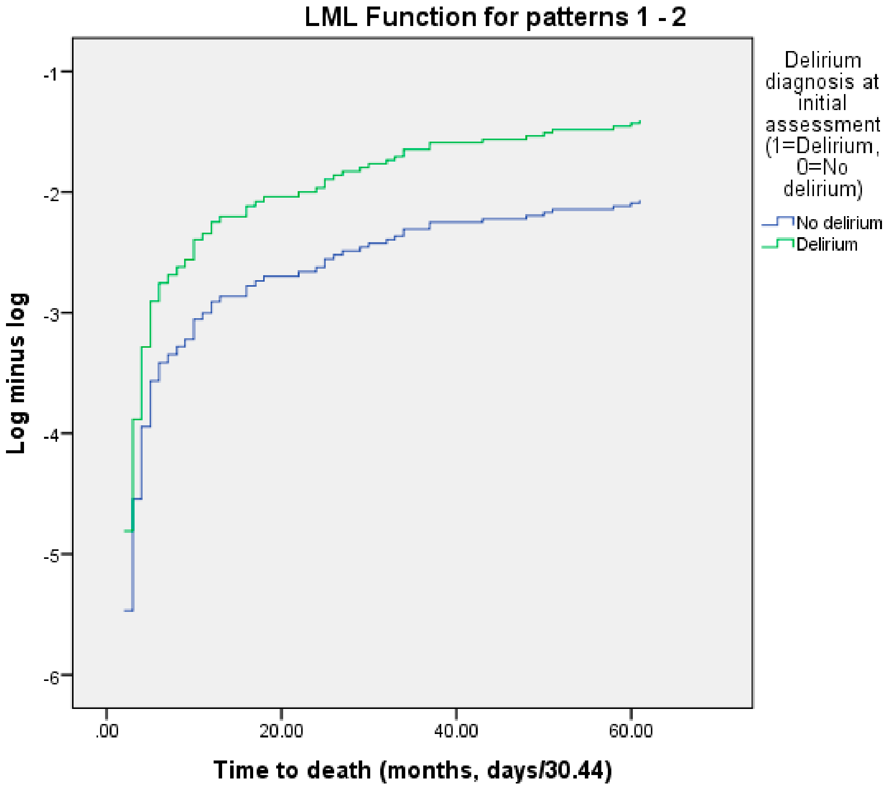

Supplementary Figure S1. Log-Minus-Log Survival Plots for Patients With and Without Delirium at Initial Assessment.

Note: The curves are plotted as log(−log(survival)) against time to death (months). The approximately parallel trajectories between groups support the proportional hazards assumption for the Cox regression model.
